# Rational Design of Disulfide Bonds Increases Thermostability of a Mesophilic 1,3-1,4-β-Glucanase from *Bacillus terquilensis*

**DOI:** 10.1371/journal.pone.0154036

**Published:** 2016-04-21

**Authors:** Chengtuo Niu, Linjiang Zhu, Xin Xu, Qi Li

**Affiliations:** 1 Key Laboratory of Industrial Biotechnology, Ministry of Education, School of biotechnology, Jiangnan University, Wuxi, China; 2 State Key Laboratory of Food Science and Technology, Jiangnan University, Wuxi, China; Universidade Nova de Lisboa, PORTUGAL

## Abstract

1,3–1,4-β-glucanase is an important biocatalyst in brewing industry and animal feed industry, while its low thermostability often reduces its application performance. In this study, the thermostability of a mesophilic β-glucanase from *Bacillus terquilensis* was enhanced by rational design and engineering of disulfide bonds in the protein structure. Protein spatial configuration was analyzed to pre-exclude the residues pairs which negatively conflicted with the protein structure and ensure the contact of catalytic center. The changes in protein overall and local flexibility among the wild-type enzyme and the designated mutants were predicted to select the potential disulfide bonds for enhancement of thermostability. Two residue pairs (N31C-T187C and P102C-N125C) were chosen as engineering targets and both of them were proved to significantly enhance the protein thermostability. After combinational mutagenesis, the double mutant N31C-T187C/P102C-N125C showed a 48.3% increase in half-life value at 60°C and a 4.1°C rise in melting temperature (*T*_m_) compared to wild-type enzyme. The catalytic property of N31C-T187C/P102C-N125C mutant was similar to that of wild-type enzyme. Interestingly, the optimal pH of double mutant was shifted from pH6.5 to pH6.0, which could also increase its industrial application. By comparison with mutants with single-Cys substitutions, the introduction of disulfide bonds and the induced new hydrogen bonds were proved to result in both local and overall rigidification and should be responsible for the improved thermostability. Therefore, the introduction of disulfide bonds for thermostability improvement could be rationally and highly-effectively designed by combination with spatial configuration analysis and molecular dynamics simulation.

## Introduction

1,3–1,4-β-glucanases (1,3–1,4-β-D-glucan 4-glucanohydrolase; EC 3.2.1.73) can hydrolyze high molecular weight β-glucans into oligosaccharides and they are widely applied in beer brewing and animal feed industries [1]. In the brewing industry, the addition of β-glucanases in malt mashing is used to degrade the barley β-glucans, thus increasing the extract yields of malt and the filtration rate of wort [2]. In the animal feed industry, especially for broiler chicken and piglets, the addition of β-glucanases can improve the digestibility of cereal-based feed and reduce sanitary problems [3]. In both industries, the performance of β-glucanases is greatly dependent upon their thermostability and acidic pH stability. The mashing temperature in the mashing process is elevated from 48°C to 78°C [4]. The temperature in animal-feed pelleting is usually between 60°C and 90°C before cooling and the digesting temperature of animals is about 40°C [5]. The 1,3–1,4-β-glucanases need to keep high activity in wide range of temperatures. Some thermophilic 1,3–1,4-β-glucanases holding high optimal temperatures (like 80–85°C) [6] cannot display high activity under the industrial conditions with relatively lower temperatures. Therefore, various mesophilic 1,3–1,4-β-glucanases are subjected to increase the thermostability [7]. Moreover, the pH of malt mashing is around pH5.0–5.5 [4] and the digestive systems in livestock were also acidic [8]. Increasing the enzyme stability at low-pH environment can improve the performance of β-glucanase. Therefore, it is important to discover new thermostable β-glucanases or improve the thermostability of existing mesophilic β-glucanases by employing promising protein engineering strategies.

Engineering of disulfide bonds in proteins is a common strategy in rational design, which has been applied to improve the thermostability of various enzymes, such as alkaline α-amylase [9] and 1,4-β-endoglucanase [10]. Disulfide bonds could lower the total entropy of the protein, thus slowing the reversible unfolding processes of proteins [11]. They could also decrease the unfolding rate of irreversibly denatured proteins [12]. In recent years, potential residue pairs for construction of disulfide bonds can be predicted by several softwares, such as Disulfide by design (DbD) [13] and modeling of disulfide bonds in proteins (MODIP) [14]. However, these computational tools often predict a large amount of potential residue pairs to construct disulfide bonds while fail to effectively identify the valid candidates. Therefore, more information is required to precisely select the residue pairs and rationally design the disulfide bonds to improve the protein thermostability.

In our previous works, a new 1,3–1,4-β-glucanase from *Bacillus terquilensis* CGX 5–1 was cloned and expressed in *Escherichia coli* BL21(DE3) [15]. Its thermostability and catalytic efficiency were enhanced through chemical modification [16] and lysine-based site-directed mutagenesis [7]. In the present study, the contribution of disulfide bonds to the thermostability of 1,3–1,4-β-glucanase was analyzed by introducing two new disulfide bonds designed by spatial configuration analysis and computational prediction. The effect of disulfide bonds on protein overall/regional flexibility were calculated by MD simulation. Additional combinational mutation based on single mutations was constructed. Besides thermostability, other biochemical properties, including pH stability and catalytic efficiency, of the mutants were also characterized and compared with wild-type enzyme. To our knowledge, this was the first study which improved the thermostability of 1,3–1,4-β-glucanases by disulfide bond engineering.

## Materials and Methods

### Strains, vectors and culture condition

The β-glucanase gene *BglTM* was designed and constructed in our previous study [7]. The restriction enzymes (*Bam*HI and *Xho*I) and the vector pMD19T-simple were purchased from Takara (Dalian, China). The vector pET28a(+) (Invitrogen, Shanghai, China) and *E*. *coli* BL21(DE3) cells were used for recombinant plasmid construction and expression. The 5,5’-Dithiobis-(2-nitrobenzoic acid) (DTNB) and isopropyl-β-D-1-thiogalactopyranoside (IPTG) were obtained from Sangon (Shanghai, China). The substrate AZO blue barley β-glucan was obtained from Megazyme (Wicklow, Ireland). The Ni-NTA column for protein purification was purchased from Qiagen (Shanghai, China) while the PD-10 desalting column was obtained from GE healthcare (Tianjin, China). Other reagents were purchased from Sinopharm (Shanghai, China).

### Cloning and expression of wild-type BglTM and its mutants

Site-directed mutagenesis of *BglTM* gene was performed using overlapping extension PCR [7] with pET28a(+)-*BglTM* plasmid as template DNA. The primers used in this study are listed in S1 Table. The PCR products were purified, ligated into vector pET28a(+) and transformed into *E*.*coli* JM109 competent cells. Positive plasmids were verified by DNA sequencing (BGI, Shanghai, China) and finally transformed into *E*.*coli* BL21(DE3) competent cells.

The transformants harboring the mutant genes were incubated in LB media at 37°C, 200 rpm for 11 h and then transferred into optimized TB medium (20 g Yeast Extract/L; 12.5 g Trypton/L; 14.1 mL Glycerol/L; 2.17 g KH_2_PO_4_/L and 2.74 g K_2_HPO_4_/L) for continuous cultivation. When the optical density at 600 nm reached 1.0, IPTG (0.06 mM) and α-lactose (8 mM) were simultaneously added to induce protein expression. After induction at 24°C for 6 h, the cells were harvested by centrifugation at 9000×g, 4°C for 20 min. The target protein was purified by a 5 cm×1 cm^2^ Ni-NTA affinity column following the procedures described in our previous study [16]. The obtained solution was then desalted using the PD-10 desalting column (GE healthcare, Tianjin, China) and concentrated using a Pellicon Cassette concentrator (Millipore, Darmstadt, Germany) with a 10 kDa cutoff membrane. The protein purity was verified by sodium dodecyl sulfate-polyacrylamide gel electrophoresis (SDS-PAGE) analysis. The protein concentration was measured according to Bradford method [17] using bovine serum albumin (BSA) as the standard protein.

### Verification of disulfide bonds formation

The formation of disulfide bonds in wild-type BglTM and its mutants was examined using DTNB which could quantitatively determine the number of free sulfhydryl groups in protein structure [18]. Two protein samples (1 mg/mL) dissolved in 20 mM phosphate buffer (pH6.5) were incubated at 37°C for 2 h, where one sample was treated with DTT at a final concentration of 100 mM while the same amount of 20 mM phosphate buffer (pH6.5) was added to the other sample. Then the two samples were dialyzed against 20 mM phosphate buffer (pH6.5) overnight to completely remove the DTT. At the end of dialysis, the protein samples were concentrated by Pellicon Cassette concentrator and the protein concentration was determined. 1 mL DTNB solution (2 mM) was mixed with 500 μL reduced or non-reduced protein samples and the mixtures were incubated at 37°C for 10 min. Absorbance at 412 nm was measured to monitor the subsequent release of the aromatic thiolate. Acetyl cysteine was used to make the standard curve. Each measurement was independently repeated in triplicate.

### Assay of 1,3–1,4-β-glucanase activity and kinetic parameters

The activity of 1,3–1,4-β-glucanase was characterized as the release of reducing sugar during hydrolysis of barley β-glucan and measured using an improved AZO method as described previously [15]. Each measurement was repeated in triplicate. The kinetic parameters (*K*_m_, *V*_max_, *k*_cat_ and *k*_cat_/*K*_m_) of wild-type BglTM and its mutants were determined by measuring the enzymatic activities at 40°C and pH6.5 while the substrate concentration was varied from 0–10 mg/mL. The values of *K*_m_ and V_max_ were estimated by Lineweaver-Burk double reciprocal method [19]. The enzyme concentration was set at 100 μg/mL.

### Effects of pH and temperature on enzymatic activity

To predict the protein kinetic stability, the optimal temperatures of the purified enzymes were determined by measuring the enzymatic activity at various temperatures at intervals of 5°C from 35°C to 75°C. For each enzyme, the highest activity measured was designated as 100%. To obtain the temperature of half-inactivation (*T*_50_) values, the purified enzymes were incubated in thermostatic water bath at 40–80°C for 10 min (the enzyme was added to preheated solution). The *T*_50_ value was defined as the temperature when the enzyme lost half of its activity after the above treatment. The half-life values of the purified enzymes were determined by incubating the enzyme at selected temperatures and measuring the enzymatic activity at a series of time points. The t_(1/2,X_°_C)_ value was defined as the time when the enzymatic activity declined to its half at a set temperature. The protein melting temperature (*T*_m_), which is an important parameter for protein thermodynamic stability, was determined by a Q2000 differential scanning calorimeter (DSC) (TA, New castle, USA) at a protein concentration of 1 mg/mL in 20 mM phosphate buffer (pH6.5). To prevent the protein aggregation at high temperatures, 1.5 M guanidine hydrochloride (Gdn/HCl) was added into the enzyme solution as previously reported [20]. The temperature was increased from 25°C to 90°C at a scan rate of 1°C/min.

To estimate the optimal pH, the purified enzymes were incubated in the following pH buffers: 20 mM HAc-KAc buffers (pH4.5–5.5) and 20 mM phosphate buffers (pH6.0–8.0). The highest activity measured was considered as 100%. For pH stability, the purified enzymes were incubated in the above buffers at 40°C for 1 h and the enzymatic activities were measured at pH6.5 and 40°C. The activities of enzymes without treatment were designated as 100%.

### Circular dichroism (CD) analysis

The far-UV CD spectra of wild-type BglTM and its mutants were recorded using MOS-45 AF circular dichroism spectrometer (Bio-logic) as described previously [7]. The Dichroweb online software [21] was used to estimate the percentages of secondary structures (helix, β-sheet, β-turns, and loops).

### MD simulation

The 3D structure of BglTM was homologically modeled in SWISS-MODEL online server [22] with the 3D structure of β-glucanase from *B*. *subtilis* (PDB code: 3O5S) [23] as template. The amino acid sequence identity between BglTM and 3O5S is 90.65% (S1 Fig). The 3D structures of BglTM and its mutants designed by DbD version 1.20 were all checked using pymol software before subjection to MD simulation processes. The ligands and solvent molecules were removed. The GROMOS 53a6 force field [24] in GROMACS 4.6.5 package was applied on a LINUX architecture. The proteins were put in cubic boxes which were filled with explicit SPC/E water molecules [25]. The distances between the edge of the boxes and the nearest protein atoms were no less than 15 Å. After charge neutralization by adding sodium ions, the system went through three steps of energy minimization process using steepest descent algorithm and conjugated-gradient algorithm. The total energy was finally converged to 10 kJ/min·nm. Periodic boundary conditions were applied and the long-range electrostatic interactions were calculated by Particle Mesh Ewald (PME) method [26]. The non-bonded van de Waals interactions were calculated by L-J model with a cut-off distance of 14 Å. The system temperature was gradually heated to the desired temperatures using a canonical NVT ensemble, followed by the equilibrium at 1 bar using a canonical NPT ensemble. Berendsen algorithm [27] and Parrinello-Rahman algorithm [28] were used to maintain the temperatures and pressure at the set level. Then the MD simulation was carried out for 20 ns at 500 K to accelerate the unfolding rate of β-glucanase based on the assumption that the unfolding pathway was not changed at high temperatures while the rate was greatly accelerated [29,30]. The atomic trajectories were saved every 4 ps. All molecular structures were visualized by pymol software version 1.5.0.3. The calculation of ionic interactions in the enzymes was carried out by Protein Interactions Calculator online server [31].

## Results

### Selection of potential residue pairs of BglTM to improve thermostability

In this study, a total of 28 potential residue pairs were predicted to meet the geometric parameters for the formation of disulfide bonds excluding the native one (C32-C61) in BglTM by DbD software (S2 Table). To reduce the number of potential residue pairs, the spatial configuration of each predicted residue pair was examined and those which would negatively affect the native structure were excluded. For example, the predicted residue pair C32-F59C was conflicted with the native disulfide bond (C32-C61). Six predicted residue pairs (G23C-Y24C, S55C-K58C, P81C-S89C, G96C-W103C, D150C-K157C and G159C-L164C) were further removed because the distance of two residues in primary sequence was less than 10 amino acids, which might produce the potential structural confliction in native secondary structure[32,33]. Upon further analysis, seven predicted residue pairs (Y72C-W151C, P81C-F110C, S89C-F110C, S89C-A204C, S90C-E109C, T93C-G177C and D104C-P173C) which located within 5Å distance of the catalytic center (Glu105 and Glu109) were also excluded to ensure the integrity of the catalytic center. Since there were still too many residue pairs, addition selection criteria were necessary.

The effect of the remaining 14 residue pairs on protein overall and local flexibility was characterized. As shown in Table 1, 11 out of 14 predicted residue pairs showed decreased root-mean-square deviation (RMSD) values in engineered regions (within 5Å distance of the mutant sites) compared to wild-type BglTM. Among them, four residue pairs (G3C-Q68C, N31C-T187C, K83C-A141C and P102C-N125C) showed the biggest drop in local flexibility. These four residue pairs also linked the regions with the highest mobility indicated by the sum of root-mean-square fluctuation (RMSF) values (S3 Table). The changes in enzyme overall flexibility were also determined (Table 2). The overall flexibility of β-glucanases was almost unchanged when most of the disulfide bonds were introduced. The addition of two disulfide bonds (N31C-T187C and P102C-N125C) greatly decreased protein flexibility while the introduction of residue pair K83C-A141C increased the protein overall flexibility.

**Table 1:** Comparison of the average RMSD values in the mutant regions (within 5Å distance from the disulfide bonds) between wild-type BglTM and the mutants.

Ranking	Residue pairs	Average RMSD value within 5Å distance from the disulfide bonds (nm)		
		WT	Mutants	RMSD_mutant_-RMSD_WT_
1	N31C-T187C	0.676	0.434	-0.242
2	K83C-A141C	0.710	0.519	-0.191
3	P102C-N125C	0.528	0.389	-0.139
4	G3C-Q68C	0.784	0.650	-0.134
5	A82C-L202C	0.671	0.585	-0.086
6	L49C-G62C	0.542	0.462	-0.08
7	R65C-M180C	0.562	0.489	-0.073
8	I87C-N185C	0.756	0.710	-0.046
9	E76C-R210C	0.641	0.597	-0.044
10	G86C-T196C	0.751	0.718	-0.033
11	R78C-T146C	0.772	0.768	-0.004
12	T95C-G177C	0.524	0.534	0.01
13	D22C-A36C	0.527	0.555	0.028
14	E63C-N182C	0.541	0.575	0.034

**Table 2:** Comparison of the average overall RMSD values between wild-type BglTM and the mutants.

Ranking	Residue pairs	Average overall RMSD (nm)		
		WT	Mutants	RMSD_mutant_-RMSD_WT_
1	N31C-T187C	1.214	1.111	-0.103
2	P102C-N125C		1.116	-0.098
3	R65C-M180C		1.191	-0.023
4	R78C-T146C		1.196	-0.018
5	L49C-G62C		1.197	-0.017
6	G86C-T196C		1.2	-0.014
7	A82C-L202C		1.203	-0.011
8	I87C-N185C		1.207	-0.007
9	E76C-R210C		1.234	0.02
10	G3C-Q68C		1.236	0.022
11	T95C-G177C		1.242	0.028
12	D22C-A36C		1.26	0.046
13	K83C-A141C		1.277	0.063
14	E63C-N182C		1.29	0.076

Based on the above analysis, two residue pairs (N31C-T187C and P102C-N125C) which could lower both the protein overall and local flexibility were chosen as candidates for engineering. To better elaborate the relationship between protein overall/local flexibility and protein thermostability, two mutants harboring the residue pairs G3C-Q68C and K83C-A141C with decreased local flexibility and increased overall flexibility were also constructed.

### Expression and disulfide bond determination of mutant enzymes

The 4 mutants with residue pairs (G3C-Q68C, N31C-T187C, K83C-A141C and P102C-N125C) were constructed, expressed in *E*. *coli* BL21(DE3) and purified (S2 Fig). The formation of the disulfide bonds in recombinant mutants was verified by DTNB method. Four free sulfhydryl groups were detected in each mutant under reducing condition and no free sulfhydryl groups was detected under non-reducing conditions (S4 Table), which meant that the new disulfide bonds were formed in the mutants.

### Thermostability analysis of wild-type BglTM and the mutants

**Fig 1:**
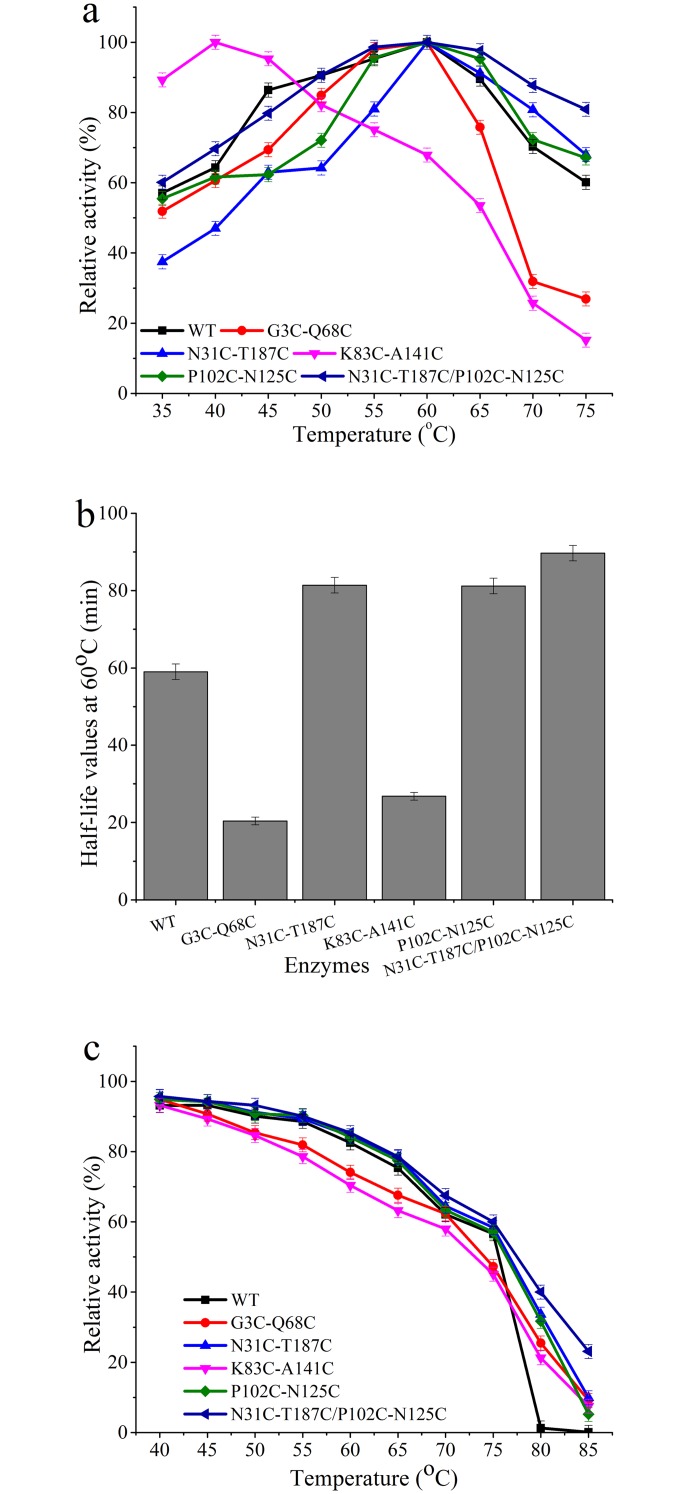
The optimal temperatures (a), enzyme inactivation curves at 60°C (b) and kinetic stability curves (c) of wild-type BglTM and the mutants.

The kinetic and thermodynamic stability of wild-type BglTM and the four mutants were determined and compared. As we can see from Fig 1a, three mutants (G3C-Q68C, N31C-T187C and P102C-N125C) displayed similar activity profiles under different temperature conditions compared to wild-type BglTM. Meanwhile, the optimal temperature of K83C-A141C mutant dropped from 60°C to 40°C. The N31C-T187C and P102C-N125C mutants showed an improvement in kinetic stability, since their half-life values at 60°C were extended from 59 min to 81.4 min and 81.2 min, respectively. The t_(1/2, 60_°_C)_ values of G3C-Q68C and K83C-A141C mutants were decreased (Fig 1b). The changes in enzyme kinetic stability were further confirmed by the half-inactivation (*T*_50_) values. The *T*_50_ values of N31C-T187C and P102C-N125C mutants were 77.1°C and 76.8°C, which were 1.1°C and 0.8°C higher than that of wild-type BglTM, while the other two mutants showed lower *T*_50_ values (Fig 1c). As for protein thermodynamic stability, the *T*_m_ values of N31C-T187C and P102C-N125C mutants were 52.2°C and 53.1°C, respectively, which were 1.4°C and 2.3°C higher than that of wild-type BglTM, while the other two mutants showed lower *T*_m_ values (Table 3). The DSC results also revealed several protein thermodynamic parameters including enthalpy change (ΔH) values, entropy change (ΔS) values and free energy (ΔG) values. As we can see from Table 3, the ΔH values of N31C-T187C and P102C-N125C mutants were increased as the *T*_m_ values were increased, which showed that more energy were needed for denaturation of the mutants. The ΔG values for these two mutants were 0.5 kcal·mol^-1^ and 0.7 kcal·mol^-1^ higher than that for wild-type BglTM. These results indicated that the addition of two disulfide bonds (N31C-T187C and P102C-N125C) could enhance the enzyme kinetic and thermodynamic stability while the other two disulfide bonds decreased enzyme thermostability. Since beneficial mutagenesis might have an additive effect on enzyme thermostability [34,35], the double mutant (N31C-T187C/P102C-N125C) containing these two positive disulfide bonds was constructed. After expression and purification, the formation of two new disulfide bonds in N31C-T187C/P102C-N125C mutant was confirmed by DTNB method (S4 Table). The optimal temperature of N31C-T187C/P102C-N125C mutant remained 60°C while its *T*_50_ value was 77.5°C, which was 1.5°C higher than that of wild-type BglTM. Its half-life value at 60°C was further extended from 59 min to 87.5 min (Fig 1). The double mutant also showed better thermodynamic stability. Its *T*_m_ value was 54.9°C, which was 4.1°C higher than that of wild-type BglTM. Meantime, its free energy value was 10.3 kcal·mol^-1^, which was 1.5 kcal·mol^-1^ higher than that of wild-type BglTM (Table 3).

**Table 3:** Thermodynamic parameters of wild-type BglTM and the mutants.

	*T*_m_ (°C)	Δ*H* (kcal·mol^-1^)	ΔG (kcal·mol^-1^)
WT	50.8	189.4	8.8
G3C-Q68C	48.7	187.6	8.4
N31C-T187C	52.2	201.9	9.3
K83C-A141C	46.9	167.2	8.1
P102C-N125C	53.1	197.3	9.5
N31C-T187C/ P102C-N125C	54.9	207.6	10.3

To confirm the effect of newly-formed disulfide bonds on enzyme thermostability, 4 mutants (N31C, T187C, P102C and N125C) with single-Cys substitutions were characterized. The optimal temperatures of these four mutants were all 60°C while the half-life values of N31C, T187C, P102C and N125C mutants at 60°C were decreased from 59 min to 47 min, 48.9 min, 52.1 min and 37 min, respectively (S3 Fig). Therefore, the enhancement of the enzyme thermostability was resulted from the disulfide bonds formation instead of the Cys substitution.

### Effect of pH on wild-type BglTM and the mutants

**Fig 2:**
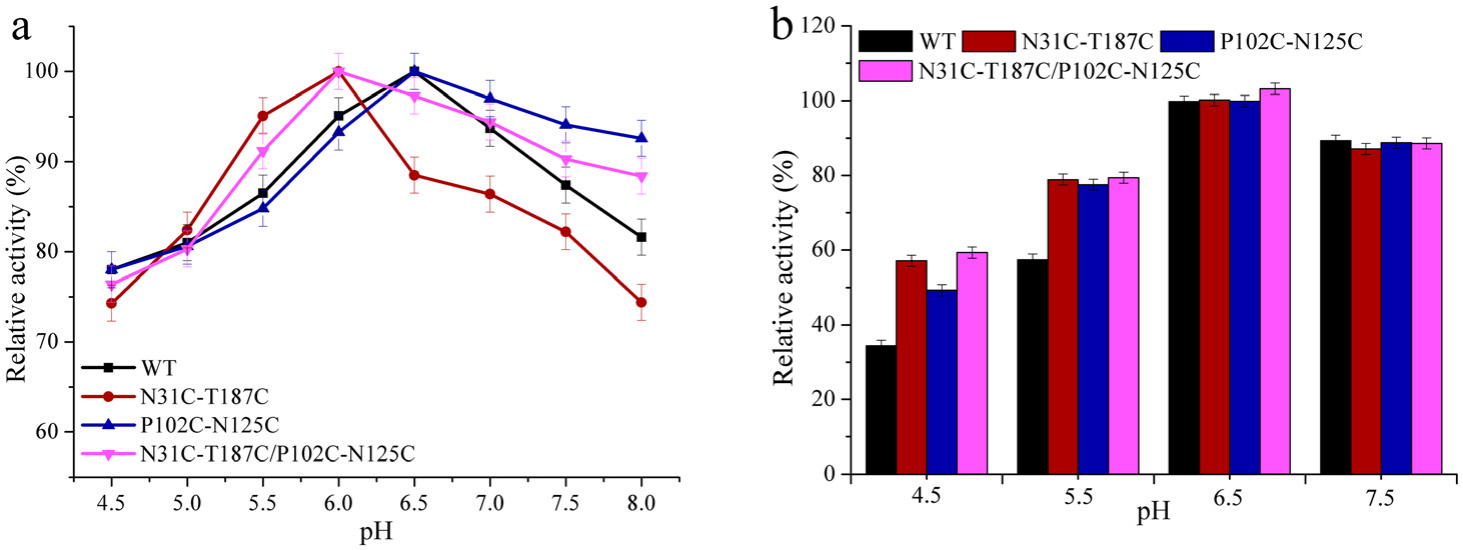
The optimal pH (a) and pH stability (b) of wild-type BglTM and the mutants.

The optimal enzymatic pH values and pH stability were compared between wild-type BglTM and the three mutants with increased thermostability. As shown in Fig 2a, the optimal pH values of wild-type BglTM and P102C-N125C mutant were determined at pH6.5, while those of N31C-T187C and N31C-T187C/P102C-N125C mutants were both dropped to pH6.0. The three mutants, especially the double mutant, also showed better stability in acidic environment (Fig 2b). There were still 61% and 79.4% activities left for N31C-T187C/P102C-N125C mutant after one hour incubation at pH4.5 and pH5.5 buffers while the remaining activities for wild-type BglTM were only 44.3% and 57.4%, respectively. The increased low-pH stability of N31C-T187C/P102C-N125C mutant was proved to result from the formation of disulfide bonds since all four mutants with single-Cys substitution displayed unaltered optimal pH and pH stability (S4 Fig).

### Catalytic properties analysis of wild-type BglTM and the mutants

The catalytic parameters of wild-type BglTM and the mutants, including the specific activities, *K*_m_, *k*_cat_ and *k*_cat_/*K*_m_, were also determined and compared (Table 4). The specific activities of N31C-T187C, P102C-N125C and N31C-T187C/P102C-N125C mutants were 4013.4±41.3 U/mg, 3998.0±30.2 U/mg and 4045.4±47.9 U/mg, respectively, which were all very similar to that of wild-type BglTM (3936.4±32.3 U/mg). Furthermore, the *K*_m_, *k*_cat_ and *k*_cat_/*K*_m_ values of the three mutants were also almost unaltered compared to those of wild-type BglTM. These results indicated that the catalytic properties of BglTM were not influenced by the formation of these two disulfide bonds.

**Table 4:** Kinetic parameters of wild-type BglTM and the mutants.

Protein samples	Specific activity (U/mg)	*K*_m_ (μM)	*k*_cat_ (s^-1^)	*k*_cat_/*K*_m_ (s^-1^·mM^-1^)
WT	3936.4±32.3	277.8±4.1	184.9±2.3	665.1±17.8
N31C-T187C	4013.4±41.3	277.3±3.4	187.6±1.9	677.3±14.2
P102C-N125C	3998.0±30.2	277.7±5.1	183.6±3.1	660.4±23.1
N31C-T187C/P102C-N125C	4045.4±47.9	277.5±4.7	186.3±2.2	672.6±17.7

### Flexibility analysis of wild-type BglTM and N31C-T187C/P102C-N125C mutant

**Fig 3:**
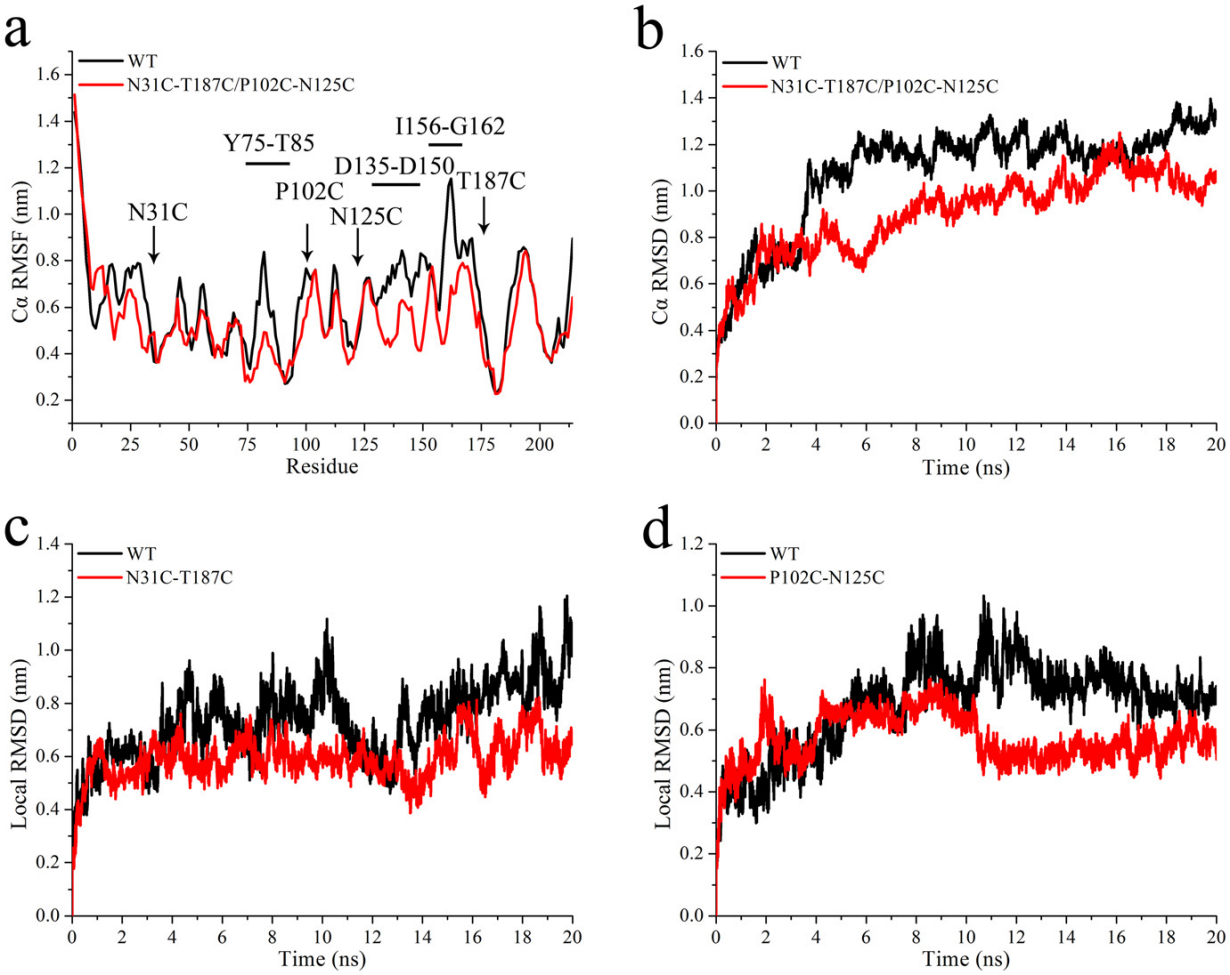
Comparison of RMSF curves (a), overall RMSD curves (b) and local RMSD curves in mutant regions N31C-T187C (c) and P102C-N125C (d) of wild-type BglTM and N31C-T187C/P102C-N125C mutant by MD simulation at 500K for 20 ns.

To understand the molecular mechanism of increased thermostability induced by the introduction of disulfide bonds, changes in protein flexibility were analyzed by MD simulation. As shown in Fig 3a, the RMSF values around the four mutation sites of N31C-T187C/P102C-N125C mutant were reduced compared to those of wild-type BglTM. Besides, RMSF values in three other regions (Y75 to T85, D135 to D150 and I156-G162) were also dropped. A more detailed description of the protein behavior at high temperatures was shown by the overall and local flexibility. The overall Cα RMSD value of wild-type BglTM stabilized around 1.214 nm at 500K, while the RMSD value of N31C-T187C/P102C-N125C mutant balanced around 1.046 nm (Fig 3b). Fig 3c and 3d showed that the average local RMSD values of N31C-T187C region and P102C-N125C region in N31C-T187C/P102C-N125C mutant were 0.612 nm and 0.589 nm, respectively, which were both slightly lower than those of wild-type BglTM (0.796 nm and 0.725 nm). These results indicated that both the overall and local structures of BglTM were rigidified by the two new disulfide bonds.

### Conformational changes in molecular structure of BglTM mutants

**Fig 4:**
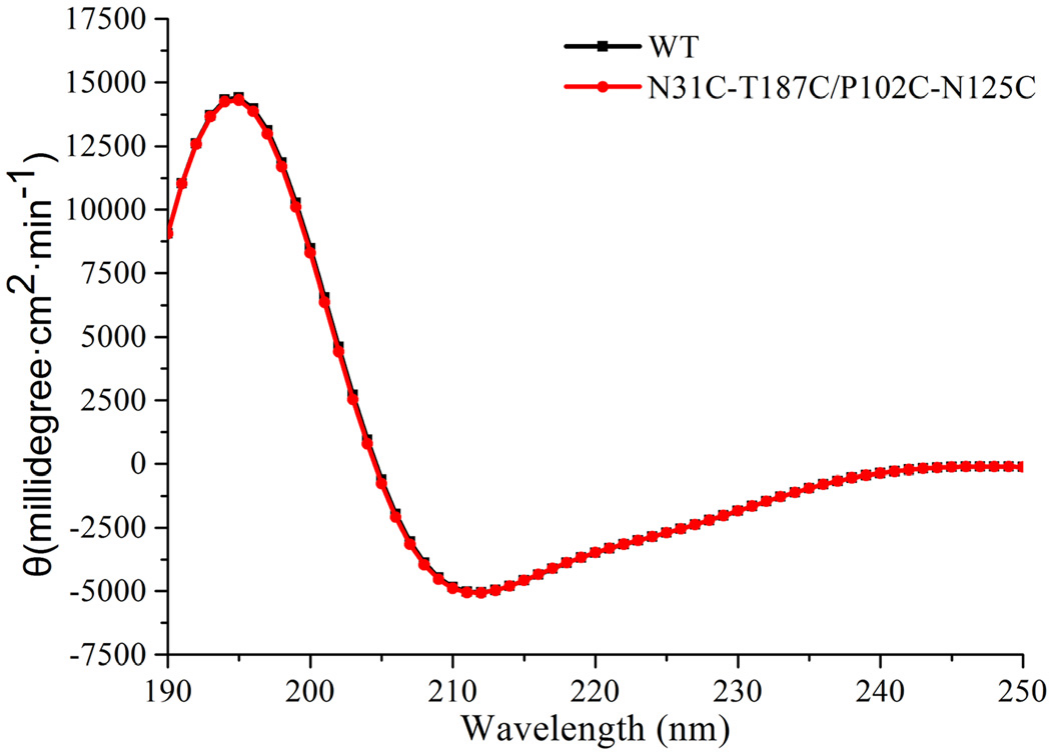
Comparison of CD spectra of wild-type BglTM and N31C-T187C/P102C-N125C mutant in 20 mM phosphate buffer (pH6.5).

To understand the mechanism of enhanced thermostability, CD analysis was applied to detect the changes in protein secondary structure. Fig 4 showed that the CD spectra of wild-type BglTM and N31C-T187C/P102C-N125C mutant were almost the same. Both enzymes displayed a peak at 195 nm and a valley around 210 nm. The results indicated that both enzymes were mainly composed of β-sheets and β-turns. The percentages of secondary structure elements (helix, β-sheet, β-turn and random coil) were estimated and no changes were observed (S5 Table). Therefore, the introduction of these two disulfide bonds did not alter the secondary structure of BglTM.

**Fig 5:**
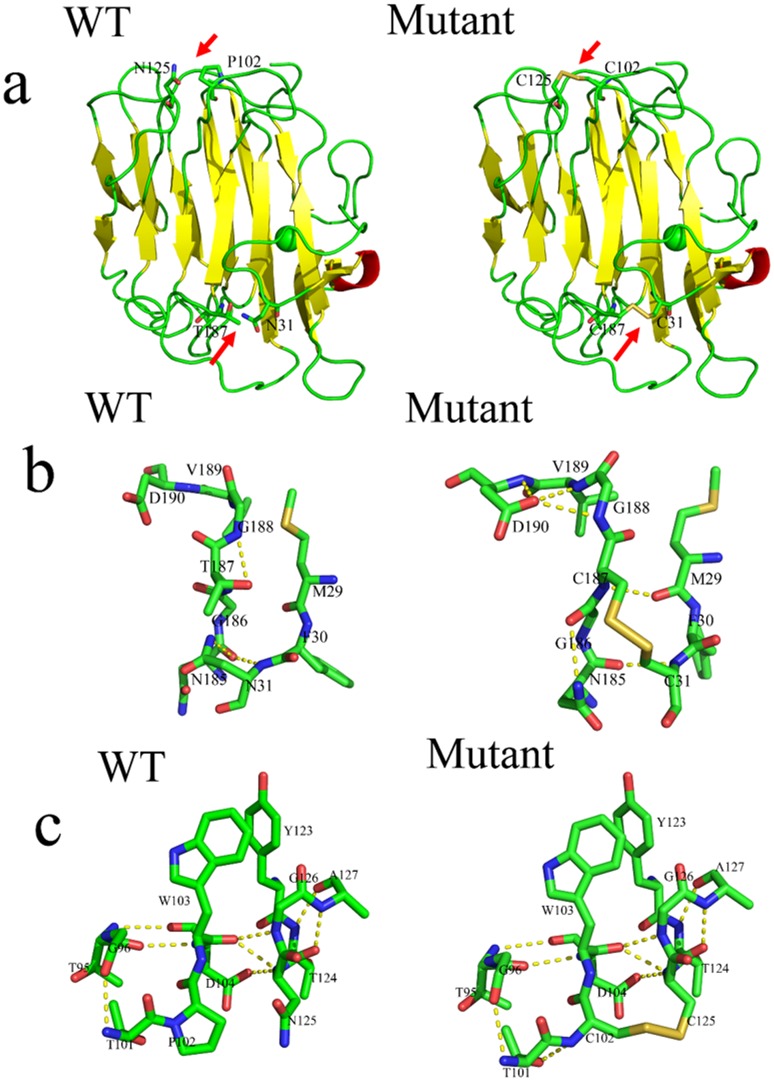
Comparison of the 3D structures of wild-type BglTM and N31C-T187C/P102C-N125C mutant (the mutant residues are shown in sticks). (a) Comparison of the overall 3D structures of wild-type BglTM and N31C-T187C/P102C-N125C mutant; (b) Comparison of the hydrogen bond network within the 5 Å region around the mutant site of N31C-T187C between wild-type BglTM and N31C-T187C/P102C-N125C mutant; (c) Comparison of the hydrogen bond network within the 5 Å region around the mutant site of P102C-N125C between wild-type BglTM and N31C-T187C/P102C-N125C mutant.

**Fig 6:**
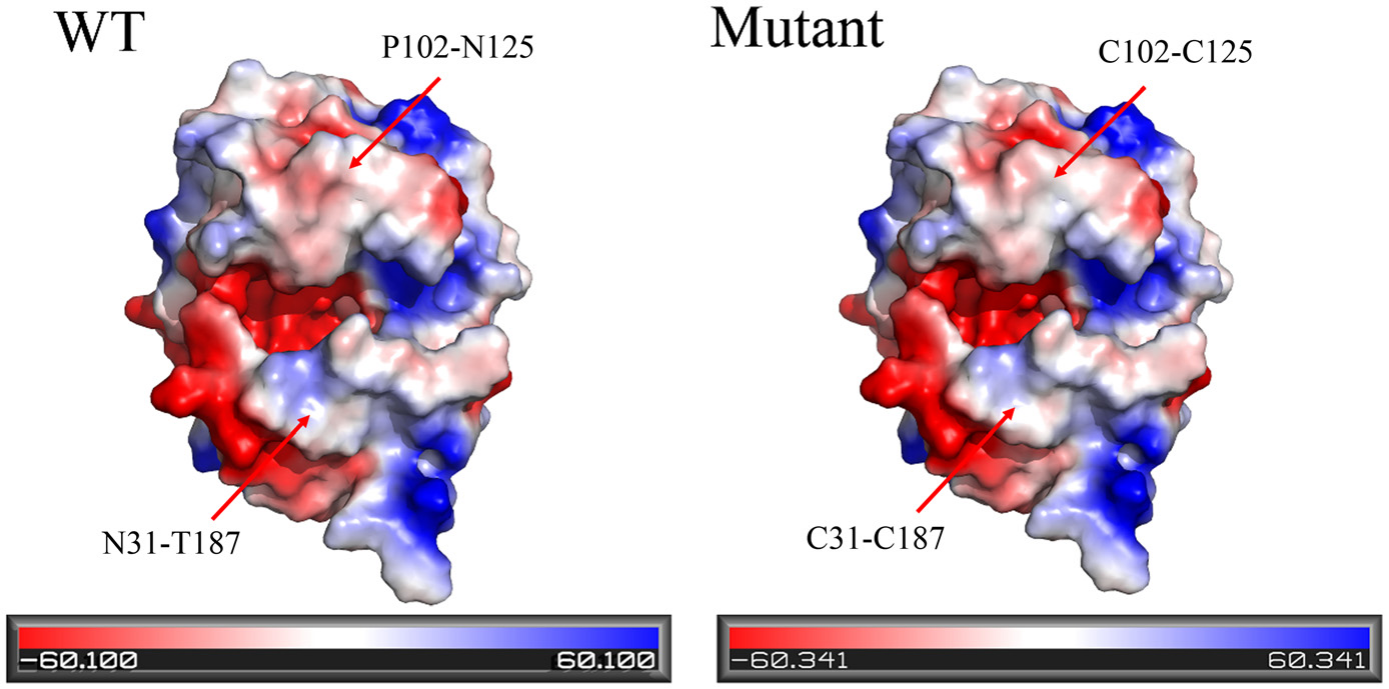
Comparison of electrostatic surface potential of wild-type BglTM and N31C-T187C/P102C-N125C mutant.

To further analyze the structural changes induced by the addition of disulfide bonds, the 3D structures of wild-type BglTM and N31C-T187C/P102C-N125C mutant were compared. As shown in Fig 5a, their overall 3D structures were similar and no changes in secondary structures were observed, which was consistent with the CD results. Two new disulfide bonds were observed in the 3D structure of N31C-T187C/P102C-N125C mutant as indicated by the red arrow. In these two mutant regions, changes in hydrogen bond network were observed. In N31C-T187C region, two native hydrogen bonds (T187-G188 and N31-G186) were lost and five new hydrogen bonds were formed, including two hydrogen bonds between D190 and V189, one between D190 and G188, one between C187 and M29 and one between C187 and N185 (Fig 5b). In P102C-N125C region, one new hydrogen bond was observed between T101 and C102 (Fig 5c). Electrostatic surface potential analysis showed that the P102C-N125C region in N31C-T187C/P102C-N125C mutant was slightly more negatively charged than the wild-type BglTM while the surface charge of other regions remained the same (Fig 6).

## Discussion

The introduction of disulfide bonds in protein structure is considered as a common strategy to improve the thermostability of industrial enzymes [36–38], however, incorrect design of disulfide bonds may also reduce the catalytic activity and thermostability [39–41]. A careful selection of the residue pairs in enzyme structure for disulfide bonds engineering was required. Previous studies showed that the disulfide bonds were often introduced in flexible regions while introducing disulfide bonds in parts of protein that are still fully structured does not contribute much to protein thermostability [42,43]. In this study, the combinational screening method considering of spatial configuration of secondary structures, catalytic center, local and overall protein flexibility proved to be high effective. Two disulfide bonds (N31C-T187C and P102C-N125C) were directly selected from 28 potential residue pairs, which enhanced the thermostability of β-glucanase. Besides, the protein secondary structure and enzyme catalytic properties were unchanged. This strategy pre-excluded the disulfide bonds which might conflict with native secondary structure and catalytic center, which could ensure the integrity of the catalytic center. The comparison of protein local and overall flexibility based on MD simulation could sort and rank the effect of potential disulfide bonds on the enzyme thermostability, and then identify the target residue pairs.

Thermostable proteins were reported to be more conformationally rigid than their mesostable homologs under high temperature condition [44,45], which meant protein thermostability might be highly correlated with its structural rigidity. Other studies also addressed the importance of local rigidity to protein thermostability [46]. The decrease of flexibility in local regions was reported to be able to rigidify the overall protein structure [47]. Besides, sometimes protein thermostability was enhanced even if there was not a decrease in global flexibility, namely potentially decrease in local flexibility were responsible for increased thermostability [44]. Therefore, simultaneously considering the changes in the flexibility of local region and overall protein is essential for the design of disulfide bonds. In this study, the addition of four residue pairs all greatly reduced the flexibility of local regions. However, only two of them (N31C-T187C and P102C-N125C) which had reduced overall flexibility enhanced the protein thermostability. The thermostability of G3C-Q68C mutant which shared similar overall flexibility with wild-type BglTM was almost unchanged while K83C-A141C mutant with greatly increased overall flexibility showed much lower optimal temperature compared to wild-type BglTM. This indicated that the decrease of local flexibility cannot ensure the rigidification of protein overall structures, which was also found in lipase B [48].

The newly-formed disulfide bonds N31C-T187C and P102C-N125C that increased the thermostability of β-glucanase were both located at the protein surface and far from enzyme catalytic center. According to the modelling 3D structure, several new hydrogen bonds were found in these two engineered regions. The increasing number of hydrogen bonds in N31C-T187C and P102C-N125C regions were three and one, respectively. These newly-formed hydrogen bonds together with disulfide bonds could rigidify the local regions and the protein overall structure, which led to the enhancement of protein thermostability [47]. The introduction of disulfide bonds in protein structure was also reported to be able to reduce the entropy of the unfolded protein, which could stabilize the protein conformation [49,50]. Therefore, the increased free energy value for the double mutant might result from a more restricted protein conformational freedom (lower entropy of unfolded protein) by the newly-formed disulfide bonds and hydrogen bonds. K83C-A141C mutant showed a great decrease in optimal temperature (form 60°C to 40°C). The reason might be the loss of a salt bridge (K83-D139) in mutant enzyme (S5 Fig), since salt bridges were reported to play important roles to rigidify enzyme structure [51,52]. This result also indicated that the salt bridge K83-D139 might be very important to the thermostability of BglTM. As for the disulfide bond G3C-Q68C locating in the most flexible regions, no increase in thermostability was observed. The disulfide bond G3C-Q68C was near the protein N-terminal, which was reported to have an important influence on β-glucanase thermostability [53,54]. The introduction of a disulfide bond in this region did not increase its thermostability.

The introduction of disulfide bonds N31C-T187C and P102C-N125C also shifted the enzyme optimal pH value from pH6.5 to pH6.0, which could enhance the performance of β-glucanases in industries. Usually, the enzyme optimal pH was affected by the protein ionizable groups [55] and surface charge [56]. Previous researches showed that acidophilic enzyme tended to have more acidic surface [57,58]. Therefore, the more negative charge in the P102C-N125C region in the double mutant might be the reason for the shift of optimal pH. The changes in pKa values of some buried ionizable groups induced by the changed interactions might also be the reason for the optimal pH shift. Furthermore, the newly formed hydrogen bonds in the mutant regions might link to the better stability in acidic conditions [9,48].

In conclusion, a high thermostable β-glucanase was obtained by introduction of two disulfide bonds (N31C-T187C and P102C-N125C) screened by protein spatial conformational analysis and MD simulation. In the meantime, the stability of β-glucanase at low-pH conditions was also improved while its catalytic property was ensured. The half-life value of N31C-T187C/P102C-N125C mutant at 60°C was extended by 48.3% while its melting temperature was increased by 4.1°C. The optimal enzymatic pH value of the double mutant was shifted from pH6.5 to pH6.0. The newly formed disulfide bonds and hydrogen bonds may contribute to the decrease of protein flexibility and lower entropy of unfolded protein, thus leading to better thermostability. This direct and rational design method combining with prediction of disulfide bonds and screening through spatial configuration analysis and molecular dynamics simulation is expected to be widely applied to engineer thermostability of other industrial enzymes.

## Supporting Information

S1 Table Nucleotide sequences of primers used in this study. (PDF)

S2 Table Residue pairs matching the geometric parameters to form disulfide bonds predicted by DbD software. (PDF)

S3 Table Ranking the sum of RMSF values of predicted residue pairs for construction of disulfide bonds in wild-type BglTM. (PDF)

S4 Table The free sulfhydryl titration of wild-type BglTM and mutants in non-reduced and reduced conditions using DTNB method. (PDF)

S5 Table Comparison of the percentage of secondary structures between wild-type BglTM and N31C-T187C/P102C-N125C mutant using Dichroweb online software. (PDF)

S1 Fig Sequence alignment of the wild-type BglTM and the reference enzyme (PDB CODE: 1GBG). (TIF)

S2 Fig SDS-PAGE gels of the purified wild-type BglTM and the mutants. (TIF)

S3 Fig The optimal temperatures (a) and protein inactivation curves at 60°C (b) of wild-type BglTM and the mutants with single-Cys substitution. (TIF)

S4 Fig The optimal pH (a) and pH stability of wild-type BglTM and the mutants with single-Cys substitution. (TIF)

S5 Fig Comparison of the 3D structures of wild-type BglTM and K83C-A141C mutant. (TIF)
