# Supplementary material for: Rational Design of Disulfide Bonds Increases Thermostability of a Mesophilic 1,3-1,4-β-Glucanase from Bacillus terquilensis
Source: PLoS One. 2016 Apr 21;11(4):e0154036. doi: 10.1371/journal.pone.0154036 (PMC4839689; doi:10.1371/journal.pone.0154036)
Supplement: S1 Table — (PDF) [file pone.0154036.s001.pdf]

S1 Table. Nucleotide sequences of primers used in this study.

Primer name[a]	Sequence (5’→3’)[b,c]	Primer name	Sequence (5’→3’)
WT-F (*Bam*HI)	cggatccatgaaacgagtgttgctaatt	T187C-R	tcatcgacacc**ACA**gccattccac
WT-R (*Xho*I)	tctcgag**G**tattttttgtatagcgcac	K83C-F	gaaaccagct**TGT**aacacaggga
G3C-F	cggctcaaaca**TGT**ggatcgttttttga	K83C-R	tccctgtgtt**ACA**agctggtttc
G3F-R	tcaaaaaacgatcc**ACA**tgtttgagccg	A141C-F	gtttgatgca**TGT**aatgcctatc
Q68C-F	aaaccgttctgtt**TGT**acatatggcta	A141C-R	gataggcatt**ACA**tgcatcaaac
Q68C-R	tagccatatgt**ACA**aacagaacggttt	P102C-F	cagatggaact**TGT**tgggatgagat
N31C-F	aaatatgttc**TGT**tgcacgtggc	P102C-R	aatctcatccca**ACA**agttccatctg
N31C-R	gccacgtgca**ACA**gaacatattt	N125C-F	actattataca**TGT**ggtgcaggaaa

T187C-F	gtggaatggc**TGT**ggtgtcgatga	N125C-R	ggtttcctgcacc**ACA**tgtataatag

[a]: the letters in brackets indicated the restriction enzymes contained in the primers; [b]: the underlined letters indicated the cutting sites of the restriction enzymes; [c]: the capital letters indicated the sites mutated.
