# Supplementary material for: Rational Design of Disulfide Bonds Increases Thermostability of a Mesophilic 1,3-1,4-β-Glucanase from Bacillus terquilensis
Source: PLoS One. 2016 Apr 21;11(4):e0154036. doi: 10.1371/journal.pone.0154036 (PMC4839689; doi:10.1371/journal.pone.0154036)
Supplement: S2 Table — (PDF) [file pone.0154036.s002.pdf]

S2 Table. Residue pairs matching the geometric parameters to form disulfide bonds predicted by DbD software.

No.	Residue No.	AA	Residue No.	AA	Chi3	Energy
1	3	GLY	68	GLN	-83.40	3.36
2	22	ASP	36	ALA	+95.88	5.73
3	23	GLY	24	TYR	+114.99	6.54
4	31	ASN	187	THR	+119.48	5.59
5	32	CYS	59	PHE	+107.08	2.77
6	32	CYS	61	CYS	+83.92	3.13
7	49	LEU	62	GLY	+86.08	3.54
8	55	SER	58	LYS	+112.23	4.34
9	63	GLU	182	ASN	+106.69	1.80
10	65	ARG	180	MET	+91.11	3.88
11	72	TYR	151	TRP	+123.65	7.21

12	76	GLU	210	ARG	+79.28	5.94
13	78	ARG	146	THR	+108.19	1.27
14	81	PRO	89	SER	+85.15	4.30
15	81	PRO	110	PHE	-84.98	2.25
16	82	ALA	202	LEU	+113.92	8.50
17	83	LYS	141	ALA	-61.05	2.99
18	86	GLY	196	TYR	-60.93	6.41
19	87	ILE	185	ASN	+109.50	2.90
20	89	SER	110	PHE	+123.19	6.15
21	89	SER	204	ALA	-99.16	3.19
22	90	SER	109	GLU	+107.66	3.06
23	93	THR	177	GLY	+124.40	2.32
24	95	THR	177	GLY	-78.62	2.59

25	96	GLY	103	TRP	+121.16	4.80
26	102	PRO	125	ASN	+100.43	5.36
27	104	ASP	173	PRO	-75.81	6.69
28	150	ASP	157	LYS	+109.45	2.96
29	159	TYR	164	LEU	-65.38	6.06
