# Supplementary material for: Rational Design of Disulfide Bonds Increases Thermostability of a Mesophilic 1,3-1,4-β-Glucanase from Bacillus terquilensis
Source: PLoS One. 2016 Apr 21;11(4):e0154036. doi: 10.1371/journal.pone.0154036 (PMC4839689; doi:10.1371/journal.pone.0154036)
Supplement: S3 Table — (PDF) [file pone.0154036.s003.pdf]

S3 Table. Ranking the sum of RMSF values of predicted residue pairs for construction of disulfide bonds in wild-type BglTM.

Ranking	Residue i			Residue j			Sum of RMSF
	Residue	Res No.	RMSF	Residue	Res No.	RMSF	
1	GLY	3	1.262	GLN	68	0.4989	1.7609
2	LYS	83	0.7054	ALA	141	0.843	1.5484
3	ASN	31	0.7395	THR	187	0.7264	1.4659
4	PRO	102	0.7193	ASN	125	0.7025	1.4218
5	GLY	86	0.5838	TYR	196	0.7208	1.3046
6	ALA	82	0.8368	LEU	202	0.4111	1.2479
7	ARG	78	0.4749	THR	146	0.7079	1.1828
8	ASP	22	0.666	ALA	36	0.3632	1.0292
9	LEU	49	0.4941	GLY	62	0.4236	0.9177
10	THR	95	0.4432	GLY	177	0.4708	0.914

11	ILE	87	0.4765	ASN	185	0.4022	0.8787
12	GLU	76	0.3355	ARG	210	0.428	0.7635
13	ARG	65	0.405	MET	180	0.2602	0.6652
14	GLU	63	0.4199	ASN	182	0.2379	0.6578
