# Supplementary material for: Rational Design of Disulfide Bonds Increases Thermostability of a Mesophilic 1,3-1,4-β-Glucanase from Bacillus terquilensis
Source: PLoS One. 2016 Apr 21;11(4):e0154036. doi: 10.1371/journal.pone.0154036 (PMC4839689; doi:10.1371/journal.pone.0154036)
Supplement: S4 Table — (PDF) [file pone.0154036.s004.pdf]

S4 Table. The free sulfhydryl titration of wild-type BglTM and mutants in non-reduced and reduced conditions using DTNB method.

Enzymes	Number of free CYS	Enzyme concentration	Expected concentration of CYS	CYS concentration based on DTNB
Non-reduced WT	0	9.475E-07 M	0	0
Reduced WT	2	9.475E-07 M	1.895E-06 M	1.734E-06 M
Non-reduced G3C-Q68C	0	7.982E-07 M	0	0
Reduced G3C-Q68C	4	7.982E-07 M	3.193E-06 M	3.001E-06 M
Non-reduced N31C-T187C	0	6.474E-07 M	0	0
Reduced N31C-T187C	4	6.474E-07 M	2.590E-06 M	2.400E-06 M
Non-reduced K83C-A141C	0	4.687E-07 M	0	0
Reduced K83C-A141C	4	4.687E-07 M	1.875E-06 M	2.012E-06 M
Non-reduced P102C-N125C	0	1.890E-06 M	0	0
Reduced P102C-N125C	4	1.890E-06 M	7.559E-06 M	7.532E-06 M

Non-reduced N31C-T187C/P102C-N125C	0	3.682E-07 M	0	0
Reduced N31C-T187C/P102C-N125C	6	3.682E-07 M	2.946E-06 M	3.064E-06 M
