# Supplementary material for: Rational Design of Disulfide Bonds Increases Thermostability of a Mesophilic 1,3-1,4-β-Glucanase from Bacillus terquilensis
Source: PLoS One. 2016 Apr 21;11(4):e0154036. doi: 10.1371/journal.pone.0154036 (PMC4839689; doi:10.1371/journal.pone.0154036)
Supplement: S5 Table — (PDF) [file pone.0154036.s005.pdf]

S5 Table. Comparison of the percentage of secondary structures between wild-type BglTM and N31C-T187C/P102C-N125C mutant using Dichroweb online software.

Protein samples	helix(%)	β-sheet(%)	β-turn(%)	loop(%)
WT	9	46	11	34
N31C-T187C/P102C-N125C	9	46	11	34
