# Supplementary figures and images for: Rational Design of Disulfide Bonds Increases Thermostability of a Mesophilic 1,3-1,4-β-Glucanase from Bacillus terquilensis
Source: PLoS One. 2016 Apr 21;11(4):e0154036. doi: 10.1371/journal.pone.0154036 (PMC4839689; doi:10.1371/journal.pone.0154036)

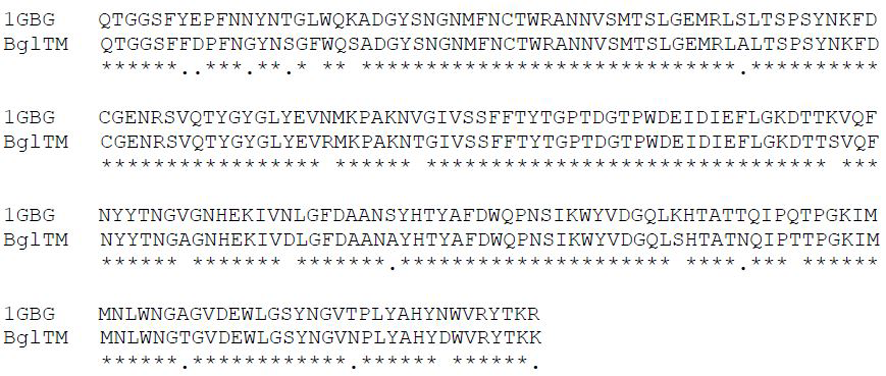

Supplement: S1 Fig — (TIF) [file pone.0154036.s006.tif]

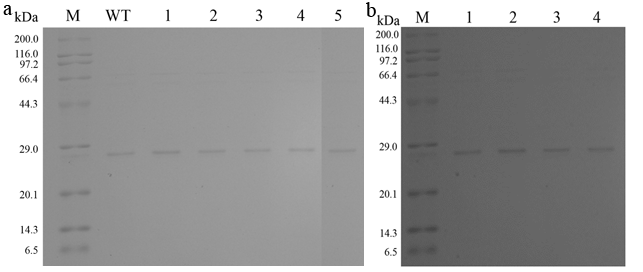

Supplement: S2 Fig — (TIF) [file pone.0154036.s007.tif]

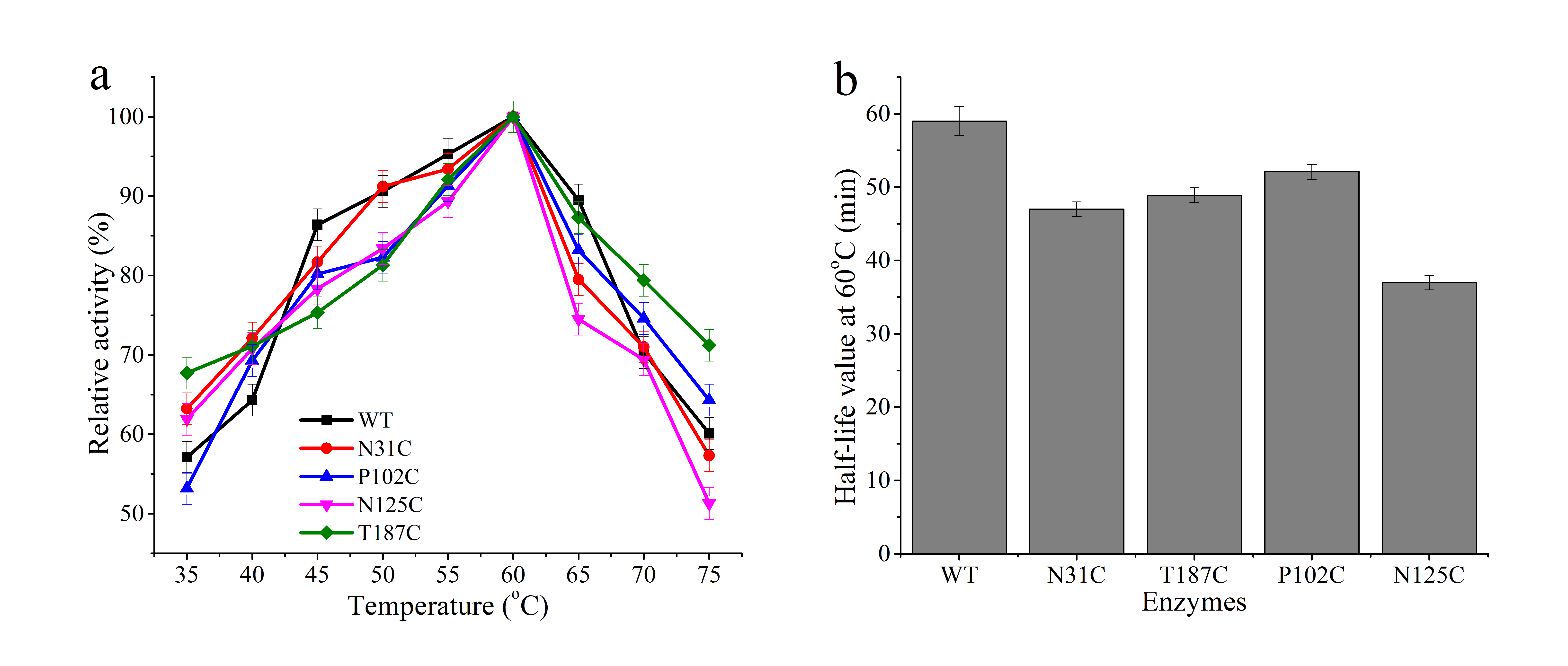

Supplement: S3 Fig — (TIF) [file pone.0154036.s008.tif]

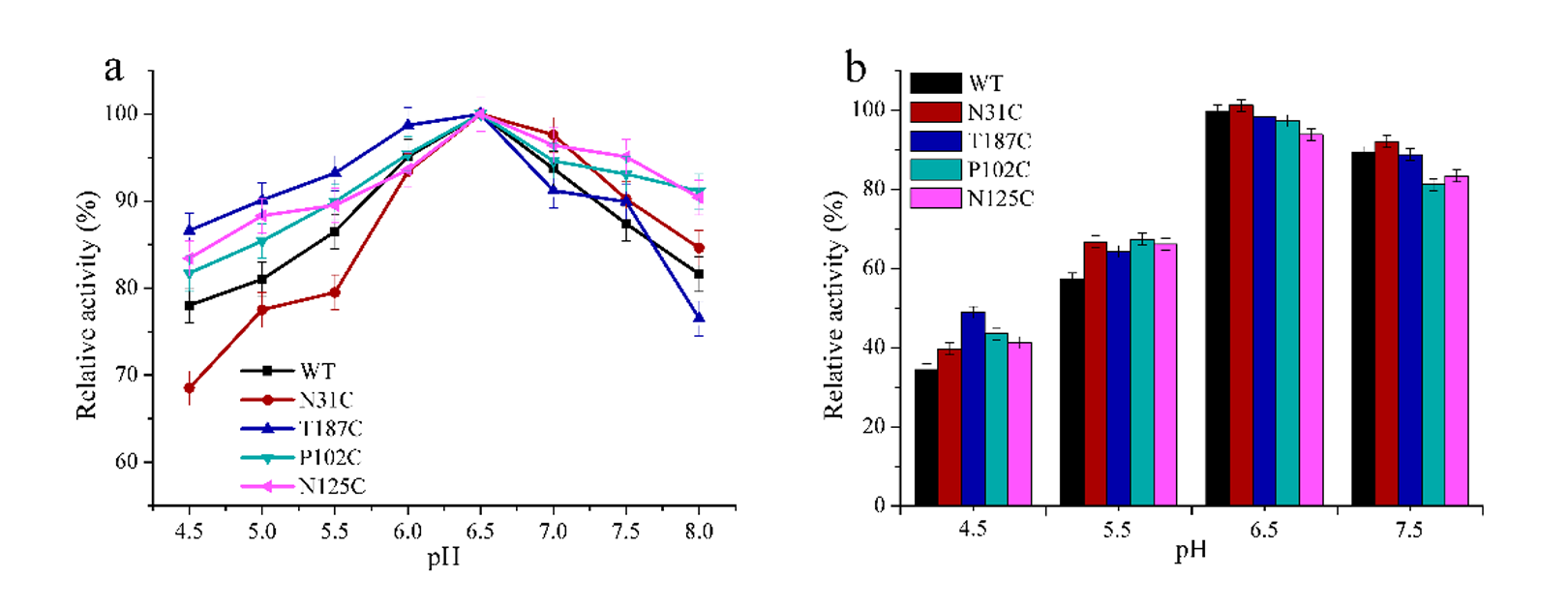

Supplement: S4 Fig — (TIF) [file pone.0154036.s009.tif]

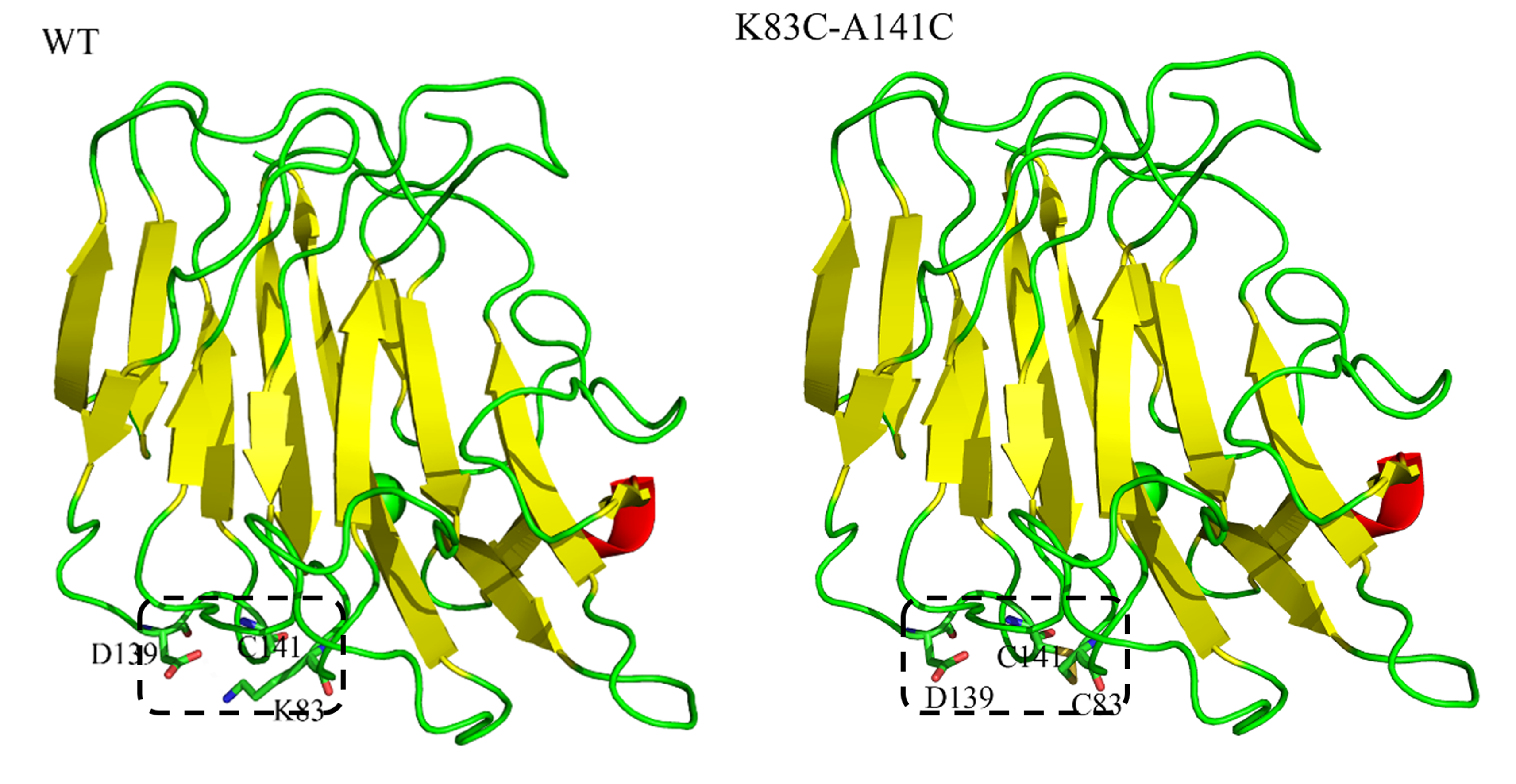

Supplement: S5 Fig — (TIF) [file pone.0154036.s010.tif]
